# Ambulatory Hypertension in Pediatric Patients With Sickle Cell Disease and Its Association With End-Organ Damage

**DOI:** 10.7759/cureus.11707

**Published:** 2020-11-25

**Authors:** Saritha Ranabothu, Michael Hafeman, Deepa Manwani, Kimberly Reidy, Kerry Morrone, Josemiguel Lorenzo, Barbara Tria, Frederick Kaskel, Joseph Mahgerefteh

**Affiliations:** 1 Pediatrics, University of Arkansas for Medical Sciences, Little Rock, USA; 2 Pediatrics, Albert Einstein College of Medicine, Bronx, USA; 3 Hematology, Children’s Hospital at Montefiore, Bronx, USA; 4 Nephrology, Children’s Hospital at Montefiore, Bronx, USA; 5 Cardiology, Children’s Hospital at Montefiore, Bronx, USA

**Keywords:** ambulatory blood pressure monitoring, end-organ complications, sickle cell disease, estimated glomerular filtration rate, hypertension, microalbuminuria

## Abstract

Background

Sickle cell disease (SCD), a chronic hemolytic disorder, results in cumulative end-organ damage affecting major organs such as the cardiovascular, renal, and central nervous systems. Effects of modifiable risk factors, such as blood pressure (BP), on the development of end-organ complications in SCD have not been well studied, particularly among the pediatric population. Relative hypertension in patients with SCD increases their risks of stroke, cardiovascular complications, and death. The primary hypothesis of this study was that abnormal BP patterns are common among patients with SCD and they impact end-organ complications.

Methods

Patients with SCD (HbSS, HbSβ0) were enrolled from the Children’s Hospital at Montefiore (N = 100). For each patient, demographic data were collected, biochemical variables in urine and blood samples were analyzed, BP was determined with ambulatory blood pressure monitoring (ABPM), and an echocardiogram was performed. The prevalence of abnormalities in BP parameters was defined, and their relationships with measures of SCD severity and end-organ damage were assessed.

Results

Sufficient ABPM data were available for 67 patients. Enrolled children were 13 ± 4 years (40% were males). Assessment of diurnal variation demonstrated that 81% of patients had abnormal systolic nocturnal dipping and 61% had abnormal diastolic nocturnal dipping. Abnormalities in the diurnal pattern were associated with reticulocytosis and hyperfiltration. Microalbuminuria was present in 19% (n = 13) of patients, of which 77% (n = 10) were females (p = 0.014). Diastolic load and abnormal nocturnal dipping were associated with hyperfiltration but not with microalbuminuria.

Conclusions

BP abnormalities detected with ABPM in SCD patients are prevalent and perhaps are a risk factor for end-organ complications. Further studies are required to identify the mechanisms underlying these relationships and their longitudinal changes.

## Introduction

Sickle cell disease (SCD) is the most common inherited disorder of red blood cells in the United States [1]. Mortality and morbidity from SCD occur due to effects on major organ systems, including cardiovascular, renal, and central nervous systems [2]. Sickle cell nephropathy is a major complication of SCD that can result in significant mortality and morbidity. It is a progressive disease with varied manifestations, such as hyperfiltration, hypertrophy, and urine-concentrating defect. Sickle cell nephropathy can progress to microalbuminuria during childhood and, by adulthood, to macroalbuminuria and decline in renal function [3].

In general, hypertension is the most important modifiable risk factor for cardiovascular and renal disease [4]. Patients with SCD are known to have lower blood pressure (BP), which is due to unexplained etiology [5], but a high prevalence of masked hypertension and abnormal circadian patterns observed from ambulatory blood pressure monitoring (ABPM) among these patients has recently gained attention [6-8]. Variability in BP and loss of nocturnal dipping of BP in patients with SCD is associated with microalbuminuria and declines in renal function in relatively small studies by other investigators [7]. Our hypothesis is that abnormal circadian patterns and masked hypertension seen on ABPM are independently related to microalbuminuria and hyperfiltration. This hypothesis was tested, and the relationship between cardiovascular complications (ejection fraction, left ventricular mass index) and BP was assessed.

## Materials and methods

Study participants and study design

Clinically stable children (age: 5-21 years; N = 100) with SCD from a pediatric sickle cell clinic at Children’s Hospital at Montefiore, Albert Einstein College of Medicine, were enrolled from November 2015 until October 2017. The study was approved by the Institutional Review Board of Children’s Hospital at Montefiore. Inclusion criteria were a diagnosis of SCD (HbSS, HbSβ0), confirmed with hemoglobin electrophoresis. Exclusion criteria were recent hospitalization, pain crisis within the prior 14 days, ongoing treatment with an antihypertensive agent, and known chronic kidney or congenital heart disease. Patients or guardians provided written informed assent/consents. Patients ≥ 18 years were allowed to consent, those 13-18 years were allowed to assent with legal guardian consent, and parents provided consent for children under the age of 13 years.

Baseline characteristics of each participant, including age, gender, weight, height, and BP, and co-morbidities such as sleep apnea, were recorded. Patients underwent 24-hour ABPM with a Spacelabs 90207 model monitor, which is compact and lightweight and has five cuff sizes available for accuracy. A trained nurse or trained pediatric nephrology fellow placed and programed ABPMs. An appropriate-sized cuff was attached to the patient’s non-dominant arm, and the device was programmed to record BP every 20 minutes during the daytime and every 30 minutes during nighttime. A study was considered interpretable when at least one BP reading per hour was valid and >25 readings in a 24-hour period were valid. The same day that an ambulatory BP monitor was placed, blood and urine samples were collected and analyzed for complete blood count, creatinine, lactate dehydrogenase (LDH), reticulocyte count, fetal hemoglobin, urine microalbumin, and urine creatinine. Estimated glomerular filtration rate (eGFR) was measured with the modified Schwartz formula for patients < 18 years old [9] and with the Modification of Diet in Renal Disease formula for patients ≥ 18 years old [10].

BP load, pre-hypertension, hypertension, and masked hypertension were defined according to the most recent ABPM statement issued by the American Heart Association [11]. BPs in the clinic were defined according to the 2017 consensus guidelines of the American Academy of Pediatrics [12].

Adequate nocturnal dipping was defined as at least a 10% decrease, relative to diurnal BP, in nocturnal systolic and diastolic BP. Blunted dipping was defined as <10% drop in nocturnal systolic or diastolic BP, extreme dipping as >20% drop in nocturnal systolic or diastolic BP, and reverse dipping as sleep systolic or diastolic BP higher than awake systolic or diastolic BP.

The systolic BP index was calculated as the measured mean BP divided by the 95th percentile for sex and height [13]. Hyperfiltration was defined as eGFR > 180 mL/min/1.73 m^2^ for patients <10 years old and as >140 mL/min/1.73 m^2^ for patients ≥10 years old [14]. Microalbuminuria was defined as urinary albumin to creatinine ratio between 30 and 300 mg/g [15].

Echocardiogram

Echocardiograms were obtained at the scheduled time annually. If echocardiogram results were more than one year old at the time of enrollment, then a repeated study was requested. All echocardiograms were performed according to the institutional protocol and in accordance with the American Society of Echocardiography published guidelines on performing and quantifying pediatric echocardiograms [16].

Data analysis

Descriptive statistics (mean ± standard deviation) were generated for all demographic characteristics. All values were checked for normality. Means, confidence intervals, standard deviations, standard errors, ranges, p-values, and equality of variances were calculated for all variables. Demographic information, anthropomorphic data, and all echocardiographic measurements and calculations were compared between patients with and without hypertension.

Pearson and Spearman correlation and multivariate regression analyses were performed. All tests were two-sided. Statistical significance was defined as p < 0.05. Statistical analyses were performed with SPSS Version 22 (IBM Corp., Armonk, NY, USA).

## Results

**Figure 1:**
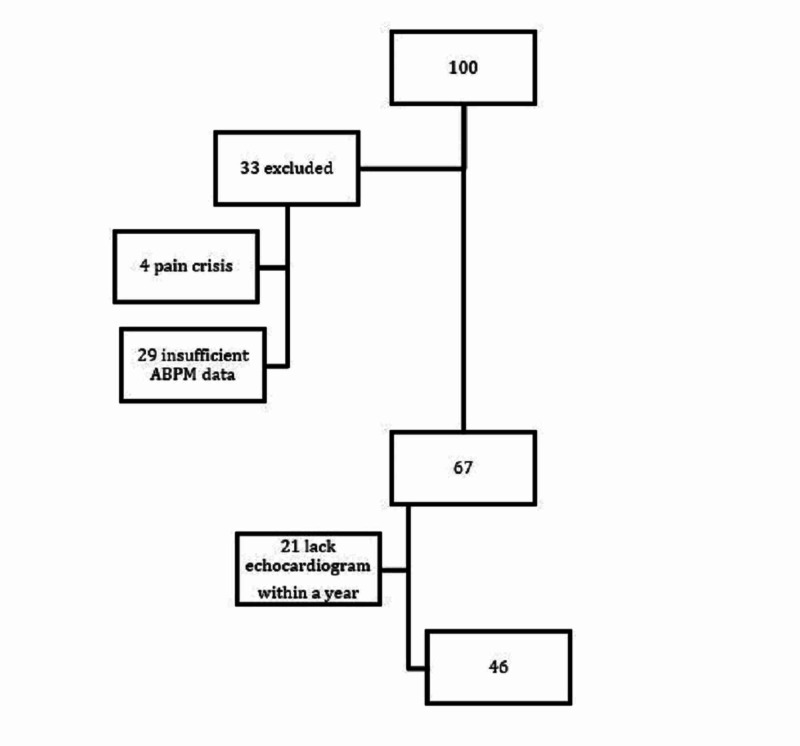
Patient cohort A total of 100 patients were enrolled; 33 patients were subsequently excluded due to pain crisis or insufficient ABPM data. Of the remaining 67 patients, 21 lacked an echocardiogram within one year of ABPM; thus, only 48 patients were included for correlation analyses of cardiovascular complications. ABPM, ambulatory blood pressure monitoring

Of 100 patients who were enrolled, 67 (40% male) were included in the analysis; the mean age was 13 ± 4 years old. Thirty-three patients were excluded due to the lack of sufficient data on ABPM (n = 29) or because a pain crisis occurred after enrollment (n = 4). Patients with no echocardiogram within one year of ABPM or insufficient echocardiogram were excluded from the cardiac analyses (n = 21) (Figure 1).

Clinical characteristics and biochemical variables are shown in Table 1. None of the patients were obese.

**Table 1:** Clinical characteristics (N = 67) BMI, body mass index; VOC, vaso-occlusive crisis (painful crisis caused by ischemic tissue injury); ACS, acute chest syndrome (acute lung injury, represents lung infarction, inflammation, atelectasis); FHx of HTN, family history of hypertension; Hb, hemoglobin; eGFR, estimated glomerular filtration rate; LDH, lactate dehydrogenase; Retic, reticulocyte; HbF, fetal hemoglobin

Age (mean ± SD)	13 ± 4 years
Sex	40% male
Race	11% Hispanic
BMI z-score (kg/m^2^)	-0.3 ± 1.3
VOC	29%
ACS	16%
Hydroxyurea	72%
Pain medications	7%
FHx of HTN	17%
Hb (g/dL)	8.7 ± 1.329%
eGFR (mL)	136 ± 38
LDH (U/mL)	468 ± 171
Urine microalbumin (mg/dL)	28 ± 46
Retic %	11 ± 6
HbF %	11.5 ± 8.6

**Figure 2:**
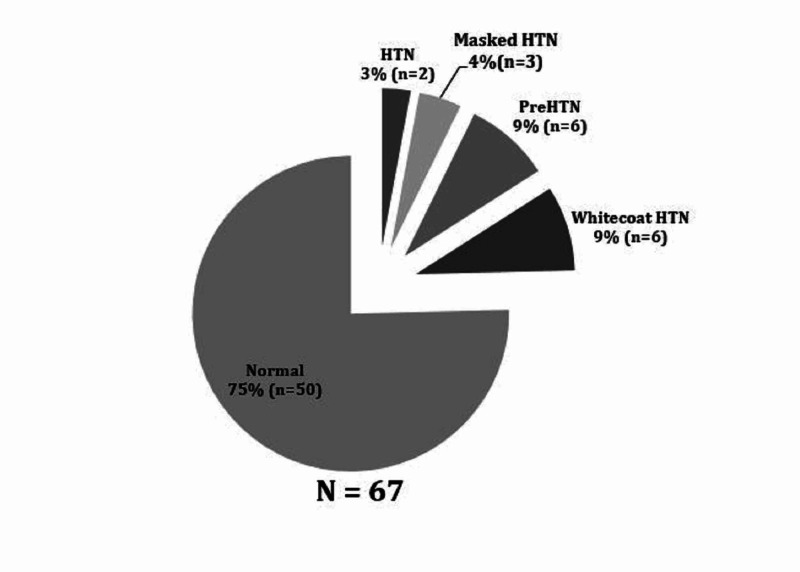
Percentage of patients with HTN HTN, hypertension

ABPM demonstrated that 7.5% (n = 5) were hypertensive (three with masked hypertension, two with ambulatory hypertension), and 9% (n = 6) were pre-hypertensive (Figure 2).

**Figure 3:**
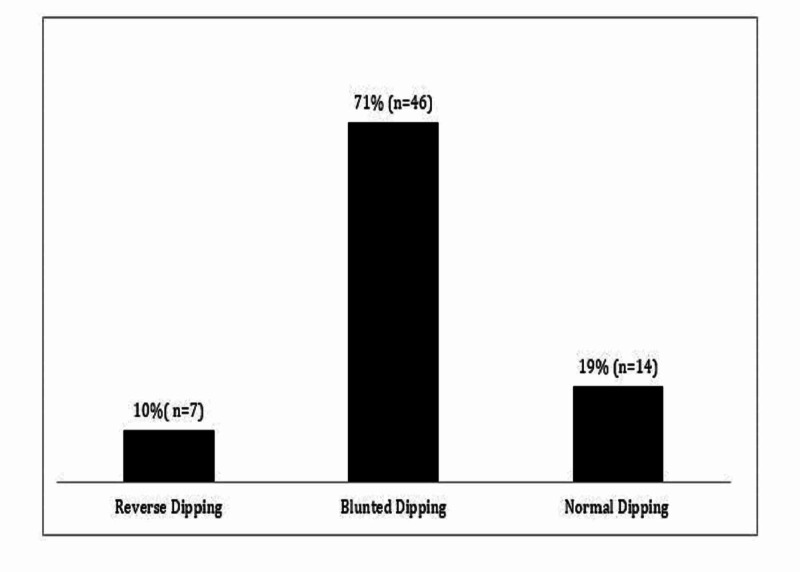
Prevalence of systolic nocturnal dipping abnormalities

Abnormal systolic nocturnal dipping was present in 81% (n = 53); of these, 10% (n = 7) had reverse systolic dipping (Figure 3).

Abnormal diastolic nocturnal dipping was present in 61% (n = 42); 9% had reverse dipping and 9% had extreme diastolic nocturnal dipping. Abnormal nocturnal dipping and hypertension were noted across all age groups. BP parameters were not associated with low hematocrit (hct), markers of inflammation (white blood cell count [WBC], absolute neutrophil count [ANC] [10,17]), or markers of hemolysis (LDH, bilirubin, aspartate aminotransferase).

Echocardiograms demonstrated that 61% of patients (30/49) had left ventricle (LV) dilation, but all had a normal systolic function, assessed by ejection fraction, and there was no evidence of significant pulmonary hypertension. Correlations between echocardiographic findings and laboratory hematologic findings are shown in Table 2.

**Table 2:** Correlations between echocardiographic findings and laboratory hematologic findings Hct, hematocrit; Retic, reticulocyte; LDH, lactate dehydrogenase; WBC, white blood cell count; ANC, absolute neutrophil count; EDV, end diastolic volume; LVMI, left ventricular mass index; LV M/V ratio, left ventricular mass-to-volume ratio

	Hct		Retic %		LDH		WBC		ANC	
	r	p-Value	r	p-Value	r	p-Value	r	p-Value	r	p-Value
EDV z-score	-0.41	<0.001	0.36	0.001	0.4	<0.001	0.3	0.006	0.13	0.24
LVMI	-0.30	0.005	0.38	0.007	0.28	0.011	0.28	0.009	0.22	0.05
LV M/V ratio z-score	0.173	0.11	-0.15	0.17	-0.13	0.22	-0.07	0.537	-0.07	0.54

As expected, results showed that left ventricular mass volume index (LVMI) and end-diastolic volume (EDV) were significantly related to hct, hemolysis (LDH), and inflammation (WBC, ANC). Correlations between ambulatory BP variables and echocardiographic markers of size and systolic function were not significant except for diastolic load, diastolic nocturnal dipping, and 24-hour diastolic BP variability (diastolic load and mitral valve S’ r = -0.3, p = 0.03; diastolic nocturnal dipping and ejection fraction z-score r = -0.2, p = 0.04; 24-hour diastolic variability and left ventricular mass-to-volume ratio z-score r = 0.3, p = 0.02; 24-hour diastolic variability and mitral valve S’ r = -0.3, p = 0.02).

Correlations between markers of renal function and laboratory hematologic findings are shown in Table 3.

**Table 3:** Correlation between hemolysis and renal function Hct, hematocrit; Retic, reticulocyte; LDH, lactate dehydrogenase; WBC, white blood cell count; eGFR, estimated glomerular filtration rate; urine MA/Cr; urine microalbumin-to-creatinine ratio

	Hct		Retic %		LDH		WBC	
	r	p-Value	r	p-Value	r	p-Value	r	p-Value
eGFR	-0.45	<0.001	0.48	<0.001	0.52	<0.001	0.26	0.01
Creatinine	0.40	<0.001	-0.45	<0.001	-0.52	<0.001	-0.26	0.01
Urine MA/Cr	-0.16	0.17	0.35	0.001	0.22	0.054	0.30	0.005

Hyperfiltration was related to hct, hemolysis (LDH), and inflammation (WBC). In addition, protein in urine was significantly related to hemolysis (LDH) and inflammation (WBC). Correlations between markers of renal function and ambulatory BP variables are shown in Table 4.

**Table 4:** Correlation between ambulatory blood pressure and renal function Sys ND, systolic nocturnal dipping; Dia ND, diastolic nocturnal dipping; 24-h sys BP var, 24-hour systolic blood pressure variability; 24-h dia BP var, 24-hour diastolic blood pressure variability; Aver HR, average heart rate; Sys load, systolic load; Dia load, diastolic load; eGFR, estimated glomerular filtration rate; urine MA/Cr, urine microalbumin-to-creatinine ratio

	Sys ND		Dia ND			24-h sys BP var		24-h dia BP var		Aver HR		% Sys load			% Dia load	
	r	p-Value	r	p-Value	r		p-Value	r	p-Value	r	p-Value		r	p-Value	r	p-Value
eGFR	-0.21	0.06	-0.25	0.02	-0.02		0.83	-0.22	0.05	0.32	0.008		0.12	0.3	0.25	0.03
Creatinine	0.12	0.30	0.15	0.19	0.07		0.55	0.25	0.02	-0.4	0.001		-0.03	0.81	-0.17	0.13
Urine MA/Cr	-0.18	0.08	-0.13	0.2	0.01		0.73	-0.03	0.94	0.05	0.72		0.06	0.64	-0.05	0.68

Hyperfiltration was related to higher diastolic load, worse nocturnal dipping, and higher average heart rate. Relationships between markers of renal function and echocardiographic findings are shown in Table 5.

**Table 5:** Correlation between renal function and echocardiogram findings EDV, end-diastolic volume; LVMI, left ventricular mass index; LV M/V ratio, left ventricular mass-to-volume ratio; EF, ejection fraction; MV S’, peak velocity of systolic mitral valve annular motion; eGFR, estimated glomerular filtration rate; urine MA/Cr, urine microalbumin-to-creatinine ratio

	EDV Z score		LVMI		LV M/V ratio Z score		EF Z score		MV S’	
	r	p-Value	r	p-Value	r	p-Value	r	p-Value	r	p-Value
eGFR	0.25	0.02	0.17	0.12	-0.22	0.05	0.23	0.04	0.02	0.84
Creatinine	-0.30	0.009	-0.11	0.31	0.23	0.04	-0.27	0.01	-0.002	0.99
Urine MA/Cr	0.02	0.81	-0.007	0.95	-0.14	0.23	0.10	0.41	0.01	0.88

Hyperfiltration was significantly related to higher left ventricular mass and lower mass-to-volume ratio. Renal function was positively correlated with left ventricular systolic function.

Microalbuminuria was present in 19% of patients (n = 13), of whom 77% (n = 10) were female (p = 0.014). Of the 28 patients who had a sleep study, eight (28.5%) were found to have sleep apnea. All but one of the patients with sleep apnea had either blunted or reverse systolic nocturnal dipping (average systolic dipping: 4.25%; diastolic dipping: 8.5%). eGFR was >90 mL/min/1.73 m^2^ for all but one of the patients; the patient was not hypertensive, did not have microalbuminuria, and had normal echocardiograms. There was no significant difference in microalbuminuria or eGFR in patients who were on hydroxyuria versus those who were not.

## Discussion

Abnormal nocturnal dipping was seen in 81% of patients; of those, 10% had reverse dipping. BP follows a circadian rhythm; levels rise during daytime or with activity and fall during nighttime or with sleep. Abnormal circadian patterns are associated with renal and cardiovascular complications in patients with chronic kidney disease or diabetes, and even within the healthy population [18-20]. Our results confirmed previous reports of a high prevalence of ABPM abnormalities, especially abnormal nocturnal dipping, in pediatric patients with SCD [6-8].

The overall rate of ambulatory hypertension of 7.42% (5/67) observed in this study population is comparable to reported prevalence in the general population of pediatric patients [21-22]. In addition, six patients were pre-hypertensive. Shatat et al. [8] reported ambulatory hypertension in 44% (n/N, 17/38) of their study population; 34% (13/38) had masked hypertension, 59% had impaired systolic nocturnal dipping, and 18% had impaired diastolic nocturnal dipping. In another study, Becker et al. obtained ABPM data on 52 pediatric patients with SCD, which showed that 35% had masked hypertension, 17% had pre-hypertension, and 56% had abnormal nocturnal dipping [7]. In the most recent study, Moodalbail et al. reported ABPM data on 56 pediatric patients with SCD that showed 30% (n = 17) were hypertensive; 3 had ambulatory hypertension and 14 had masked hypertension [6]. Half of the patients had pre-hypertension/abnormal nocturnal dipping [6]. Overall, in our study population, the prevalence of hypertension/masked hypertension was lower, but rates of blunted nocturnal dipping were higher. The higher rate of blunted dipping in our study may be due, in part, to differences in the type of SCD. For example, most patients in our study population have severe genotypes of SCD, but almost one-third of the patients in the study conducted by Shatat et al. had sickle cell trait (HbSC) [8].

Previous studies have proposed adding ABPM as a standard of care for all patients with SCD [8]. Together, our results and those from other studies support a role for 24-hour ABPM in all pediatric SCD patients and suggest that SCD may be a condition that, similar to chronic kidney disease, increases risk of masked hypertension. Importantly, the Centers for Medicare & Medicaid Services recently finalized a national coverage policy for ABPM. SCD was not specified in the policy, but it does specify that routine ABPM be considered in “children with high-risk conditions.” Similarly, SCD was not listed as a high-risk condition when the American Academy of Pediatrics recently issued its clinical practice guidelines on screening and managing hypertension in children and adolescents [12]. Our results and those of others support the inclusion of SCD as a high-risk condition.

Consistent with other studies, 19% (n = 13) of patients in our study had microalbuminuria, most of whom were females (11/13). Abnormal dipping in patients with microalbuminuria did not differ from that in patients without microalbuminuria, which is inconsistent with the results of previous studies. In concordance with previous studies, most (49%) of our study population had hyperfiltration [23]. Previous reports showed a mean eGFR of around 155 ± 40 mL/min/1.73 m^2^ (measured with 99mTc-DTPA) in children with SCD who were <16 years of age; it was shown to decline during the second decade of life, with a mean eGFR of around 133 ± 31 mL/min/1.73 m^2^ in children >16 years of age [24]. In the present study, the mean eGFR (136 ± 38 ml/min/1.73 m^2^) was lower than in the previous studies [24-25]. Of note, 28 (40%) out of 69 patients had eGFR <130 mL/min/1.73 m^2^, as determined by serum creatinine measurements. Low eGFR was not associated with high WBC, high LDH, low hemoglobin, or nocturnal hypertension, as was demonstrated in the past [26]. Hyperfiltration, however, was related to inflammation and hemolysis. Most patients (20 out of 28; 71%) with low eGFR (<130 mL/min/1.73 m^2^) were on hydroxyurea. The etiology of lower mean eGFR observed in this study, as compared to published studies, is unclear but may be related to the differences in measured 99mTc-DTPA versus calculated eGFR. Further studies are needed to establish the causal relationship and potential benefit of treating diastolic nocturnal dipping and diastolic load to slow the progression of sickle cell nephropathy.

Many studies of adults showed that SCD is associated with elevated cardiac output, cardiomegaly, cardiac dysfunction/diastolic dysfunction, and pulmonary hypertension [27-28]. In our study of a pediatric population, left ventricular dilation was seen in 61% of patients, but ventricular function was normal, there was no evidence of significant pulmonary hypertension, and systolic nocturnal dipping was not associated with cardiac remodeling. However, diastolic nocturnal dipping, diastolic load, and diastolic BP variability negatively impact left ventricular remodeling and systolic function. A previous study showed that anemia and hemoglobin concentration have a relationship with LV size [29]. As expected, our study demonstrated that cardiac size has a significant relationship with anemia, hemolysis, and inflammation. In addition, reticulocytosis was significantly correlated with ejection fraction, which may warrant further investigation.

Limitations

Conclusions drawn from the results of this study are limited by the study’s small size and cross-sectional design. All enrolled patients could not be included in the analysis because some did not have valid ABPM results or interpretable echocardiograms. Patients were included if they had at least 25 ABPM readings, which is fewer than the recommended number of measurements in the research. General limitations of ABPM that may affect measurements and detection of nocturnal abnormalities include differences between bedtime and actual sleep time, as well as discomfort due to the monitor; however, these limitations are likely to impact our study to the same extent that they impact others that have used ABPM. Data were not reproduced. Microalbuminuria was determined from a single random sample. Also, the use of creatinine to calculate eGFR may affect results. Finally, 23% of patients were on chronic transfusion, and some received ABPM on the last day of transfusion or one day after the completion of transfusion, which may affect hemoglobin and markers of hemolysis.

## Conclusions

Abnormal BP patterns were noted for patients with SCD, and accurate diagnosis of most of these abnormalities requires ABPM. Thus, the use of ABPM in this at-risk population may be warranted. SCD patients have other risk factors, such as hemolysis, vaso-occlusion, and chronic inflammation, that could contribute to end-organ complications. However, patients who are at high risk of sustained hypertension (i.e., those with pre-hypertension or masked hypertension) need to be assessed and treated carefully to lessen end-organ damage. Long-term follow-up studies are needed to assess the role of abnormal nocturnal dipping in stroke, cardiovascular, or renal complications.

## References

[1] Hassell KL (2010). Population estimates of sickle cell disease in the U.S. Am J Prev Med.

[2] Steinberg MH (2008). Sickle cell anemia, the first molecular disease: overview of molecular etiology, pathophysiology, and therapeutic approaches. ScientificWorldJournal.

[3] Nath KA, Hebbel RP (2015). Sickle cell disease: renal manifestations and mechanisms. Nat Rev Nephrol.

[4] Botdorf J, Chaudhary K, Whaley-Connell A (2011). Hypertension in Cardiovascular and Kidney Disease. Cardiorenal Med.

[5] de Jong PE, Landman H, van Eps LW (1982). Blood pressure in sickle cell disease. Arch Intern Med.

[6] Moodalbail DG, Falkner B, Keith SW, Mathias RS, Araya CE, Zaritsky JJ, Stuart MJ (2018). Ambulatory hypertension in a pediatric cohort of sickle cell disease. J Am Soc Hypertens.

[7] Becker AM, Goldberg JH, Henson M, Ahn C, Tong L, Baum M, Buchanan GR (2014). Blood pressure abnormalities in children with sickle cell anemia. Pediatr Blood Cancer.

[8] Shatat IF, Jakson SM, Blue AE, Johnson MA, Orak JK, Kalpatthi R (2013). Masked hypertension is prevalent in children with sickle cell disease: a Midwest Pediatric Nephrology Consortium study. Pediatr Nephrol.

[9] Schwartz GJ, Muñoz A, Schneider MF, Mak RH, Kaskel F, Warady BA, Furth SL (2009). New equations to estimate GFR in children with CKD. J Am Soc Nephrol.

[10] Levey AS, Bosch JP, Lewis JB, Greene T, Rogers N, Roth D (1999). A more accurate method to estimate glomerular filtration rate from serum creatinine: a new prediction equation. Modification of Diet in Renal Disease Study Group. Ann Intern Med.

[11] Flynn JT, Daniels SR, Hayman LL (2014). Update: ambulatory blood pressure monitoring in children and adolescents: a scientific statement from the American Heart Association. Hypertension.

[12] Flynn JT, Falkner BE (2017). New clinical practice guideline for the management of high blood pressure in children and adolescents. Hypertension.

[13] Samuels J, Ng D, Flynn JT, Mitsnefes M, Poffenbarger T, Warady BA, Furth S; Chronic Kidney Disease in Children Study Group (2012). Ambulatory blood pressure patterns in children with chronic kidney disease. Hypertension.

[14] Hjorth L, Wiebe T, Karpman D (2011). Hyperfiltration evaluated by glomerular filtration rate at diagnosis in children with cancer. Pediatr Blood Cancer.

[15] National Kidney Foundation (2007). KDOQI Clinical Practice Guidelines and Clinical Practice Recommendations for Diabetes and Chronic Kidney Disease. Am J Kidney Dis.

[16] Lopez L, Colan SD, Frommelt PC (2010). Recommendations for quantification methods during the performance of a pediatric echocardiogram: a report from the Pediatric Measurements Writing Group of the American Society of Echocardiography Pediatric and Congenital Heart Disease Council. J Am Soc Echocardiogr.

[17] Jodele S, Dandoy CE, Lane A (2020). Complement blockade for TA-TMA: lessons learned from a large pediatric cohort treated with eculizumab. Blood.

[18] Velasquez MT, Beddhu S, Nobakht E, Rahman M, Raj DS (2016). Ambulatory blood pressure in chronic kidney disease: ready for prime time?. Kidney Int Rep.

[19] Felicio JS, de Souza AC, Kohlmann N, Kohlmann O, Jr. Jr., Ribeiro AB, Zanella MT (2010). Nocturnal blood pressure fall as predictor of diabetic nephropathy in hypertensive patients with type 2 diabetes. Cardiovasc Diabetol.

[20] Gkaliagkousi E, Anyfanti P, Lazaridis A (2018). Clinical impact of dipping and nocturnal blood pressure patterns in newly diagnosed, never-treated patients with essential hypertension. J Am Soc Hypertens.

[21] Fan Z, Liao Z, Zong X, Zhang S (2019). Differences in prevalence of prehypertension and hypertension in children and adolescents in the eastern, central and western regions of China from 1991-2011 and the associated risk factors. PLoS One.

[22] Bell CS, Samuel JP, Samuels JA (2019). Prevalence of hypertension in children. Hypertension.

[23] Guasch A, Navarrete J, Nass K, Zayas CF (2006). Glomerular involvement in adults with sickle cell hemoglobinopathies: Prevalence and clinical correlates of progressive renal failure. J Am Soc Nephrol.

[24] Aygun B, Mortier NA, Smeltzer MP, Hankins JS, Ware RE (2011). Glomerular hyperfiltration and albuminuria in children with sickle cell anemia. Pediatr Nephrol.

[25] Bodas P, Huang A, O'Riordan MA, Sedor JR, Dell KM (2013). The prevalence of hypertension and abnormal kidney function in children with sickle cell disease -a cross sectional review. BMC Nephrol.

[26] Itano HA, Keitel HG, Thompson D (1956). Hyposthenuria in sickle cell anemia: a reversible renal defect. J Clin Invest.

[27] Fitzhugh CD, Lauder N, Jonassaint JC (2010). Cardiopulmonary complications leading to premature deaths in adult patients with sickle cell disease. Am J Hematol.

[28] Rees DC, Williams TN, Gladwin MT (2010). Sickle-cell disease. Lancet.

[29] Lester LA, Sodt PC, Hutcheon N, Arcilla RA (1990). Cardiac abnormalities in children with sickle cell anemia. Chest.

